# Aging heart and infection

**DOI:** 10.18632/aging.102128

**Published:** 2019-07-25

**Authors:** Colwyn Headley, Joanne Turner, Murugesan VS Rajaram

**Affiliations:** 1 Texas Biomedical Research Institute, San Antonio, TX 78227, USA; 2 Department of Microbial Infection and Immunity, College of Medicine, The Ohio State University, Columbus, OH 43210, USA

**Keywords:** aging, inflammaging, non-tuberculous mycobacteria, hypertrophy, fibrosis

By 2050 the elderly population will make up almost 20% of the global population, considerably increasing the number of individuals that are more susceptible to infection. Infection and infectious disease associated pathologies are often complicated by delays in immune response generation and excess inflammation that impact infection resolution. The term “inflammaging” was coined to denote the multifaceted dysregulation of homeostatic processes that over time culminates in quantifiable, organism-wide shifts towards inflammation in old age [1]. The field has rightfully dedicated a great deal of time into understanding the intrinsic (genetic predispositions, autoimmunity, chronic infections, microbiota) and extrinsic (diet, levels of physical activity/exercise, stress, air pollution) factors that contribute to inflammaging [1]. As we shift our focus towards understanding the impact of inflammaging, we have recently determined that inflammaging may also accelerate the decline in cardiovascular fitness. Aging is linked with morphological changes of the myocardium and alterations in cardiac performance including left ventricular hypertrophy, fibrosis and depressed systolic and diastolic functions in both human and animals [2]. Due to the increasingly growing elderly population, there is an urgent need for developing new therapy and understanding the molecular mechanisms of age-related cardiac diseases during infection. Only recently have we begun to elucidate the impact of bacterial infections in cardiac dysfunction in the elderly.

Age is a major prognostic factor for the development of non-tuberculous mycobacteria (NTM) disease, with recent clinical data reflecting increased incidence of NTM infection in elderly immunocompetent individuals, and NTM infection in patients with HIV, COPD, cystic fibrosis, and/or medications that modify immunity have increased disease severity. It is also known that tuberculosis (TB) caused by *Mycobacterium tuberculosis* (*M.tb*) can cause pericarditis [3], endocarditis and myocarditis leading to sudden deaths [4]. TB is a major global killer and it is estimated that 57% of all TB deaths globally occur in individuals older than 65 [5]. Based upon abundant circumstantial evidence, a direct link between mycobacterial infections, aging and cardiac dysfunction was hypothesized by our group.

We examined how mycobacterial infection and inflammaging catalyze the decline in cardiovascular function in the elderly [6].Young (3 months) and old (18 month) female C57BL/6 mice were infected with a sub-lethal dose of*, Mycobacterium avium (M. avium),* an NTM. We observed no differences in the *M. avium* bacterial numbers in the lung, liver or spleen between young and old *M. avium* infected mice. However, through the course of *M. avium* infection, old mice developed severe dysrhythmia and developed pericarditis. Moreover, the hearts of *M. avium* infected old mice had increased cardiac hypertrophy, fibrosis, expression of pro-inflammatory genes, and infiltration of immune cells, which are hallmarks of myocarditis. Since these cardiac abnormalities only manifested in old mice, we investigated several factors that contribute to this form of age dependent infectious myocarditis. Independent of *M. avium* infection, old mice had increased levels of pro-inflammatory cytokines in their serum, which may have predisposed old mice to infectious myocarditis. The reasons for increased inflammation in old age are multifaceted, and future studies will be needed to identify the principal sources of increased inflammation and whether ameliorating inflammation prevents NTM associated cardiac complications in old mice.

We and others have previously shown that virulent/pathogenic bacteria like *Streptococcus pneumonia* [7] and *Francisella novicida* [8] can invade heart tissue and subsequently lead to heart failure. Our recent work however, deviates and uses a non-pathogenic strain of mycobacteria, at relatively low (non-lethal) inoculation doses. This highlights how even low or what we may generally consider as insignificant bacterial loads can profoundly impact cardiovascular health. Our work was also the first of its kind to show on a basic level, that mycobacterial infection contributes to cardiac dysfunction in the elderly. It will be interesting to see whether similar findings arise in more virulent mycobacterial infections, such as *Mycobacteria bovis*, and *M.tb.* In conclusion *M.tb* is a significant global health concern, and the elderly are more susceptible to developing active TB disease, be it from new infection or reactivation of latent *M.tb* infection. The impact of latent mycobacteria on host cardiovascular fitness is understudied. Although the mycobacteria are considered “dormant”, the host immune exertion necessary to maintain dormancy might have a dire impact on the host.
